# An automated cell-counting algorithm for fluorescently-stained cells in migration assays

**DOI:** 10.1186/1480-9222-13-9

**Published:** 2011-10-19

**Authors:** Baraa K Al-Khazraji, Philip J Medeiros, Nicole M Novielli, Dwayne N Jackson

**Affiliations:** 1 Department of Medical Biophysics, University of Western Ontario, London, Ontario, Canada; 2 Department of Biomedical Engineering, University of Western Ontario, London, Ontario, Canada

**Keywords:** automated cell counting, threshold, migration assays, manual cell counting

## Abstract

A cell-counting algorithm, developed in Matlab^®^, was created to efficiently count migrated fluorescently-stained cells on membranes from migration assays. At each concentration of cells used (10,000, and 100,000 cells), images were acquired at 2.5 ×, 5 ×, and 10 × objective magnifications. Automated cell counts strongly correlated to manual counts (r^2^ = 0.99, P < 0.0001 for a total of 47 images), with no difference in the measurements between methods under all conditions. We conclude that our automated method is accurate, more efficient, and void of variability and potential observer bias normally associated with manual counting.

## Background

Traditionally, *in vitro *cell-counting methodologies consist of manual counts through use of a hemacytometer [1,2]. Generally, cell migration experiments are conducted using modified Boyden chambers, whereby the cells of interest migrate through a porous membrane and are stained for counting. Such migratory cells are commonly labelled on the membrane with a crystal violet stain [3,4], Trypan Blue dye [5], or hematoxylin [6-8], and quantified manually. Although it remains the gold standard, manual cell counting is very time-consuming and may introduce experimenter bias, thus increasing the potential for measurement errors [9].

In an effort to increase efficiency and mitigate potential sources of bias/error associated with manual cell counting, a number of commercially available software suites provide automated cell counting from microscopic images. These software packages enable users to collect cell counts from random fields of view within specimens of interest. Unfortunately, these software suites contain proprietary algorithms (making them inadaptable), can be expensive, and often require high performance computers.

Whole membrane quantification has been accomplished through spectrophotometric methods using absorbance microplate readers [10-12]. These microplate readers are able to detect total stained (if using dyes/stains) or fluorescence signal, where output values are a direct indication of total migrated cells. Although absorbance microplate readers allow for an expeditious analysis of migration assays, they are costly and do not provide a record of membrane images should manual confirmation or further analysis be required.

In the current study, we sought to develop a feasible, valid, and reliable algorithm designed to automate cell counting using stored images from cell migration experiments. In an effort to validate our algorithm we: 1) compared automated cell counts with manual counts from a blinded experimenter; and 2) determined the effect of objective power (2.5 ×, 5 ×, and 10 ×) and increasing the number of cells in images on our algorithm's ability to accurately resolve and quantify cells.

## Results

### Cell-Counting Algorithm

**Figure 1:**
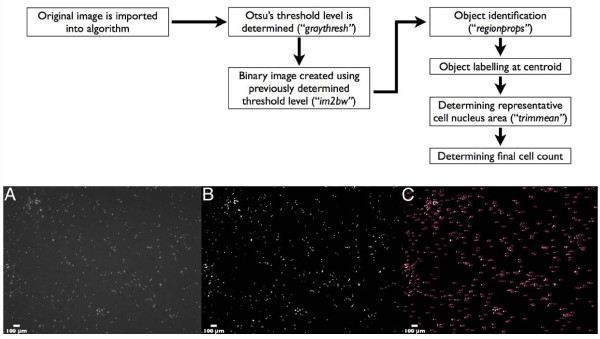
**Flow Chart of Algorithm Processes**. Panel A: original image (2.5 ×) read by algorithm; B: post-thresholding using Otsu's method for selection of threshold level. C: thresholded image with each object numerically labeled.

Matlab^®^ software was used to create an algorithm for counting 4',6-diamidino-2-phenylindole (DAPI)-stained 4T1 (murine breast cancer) cells from cell migration assays. DAPI nucleic stain provided images with high contrast between nuclei and background (Figure 1, Panel A). Our algorithm consisted of three main components (in order) for automated counting:

1) Image thresholding: Original images (Figure 1A) were read into the algorithm, and through use of the Matlab^®^ command *graythresh, *Otsu's method for global thresholding [13] was applied for selection of a threshold level used to convert the original image into a binary image. Briefly, Otsu's method segments an image by maximizing the separability of the two populations in a histogram. Three conditions must be met in order to maximize use of Otsu's method: a) minimum variability in grey levels of foreground objects, b) minimum variability in background grey levels, c) maximum variability between background and foreground objects grey levels. These conditions are met by the DAPI-nucleic stained images used in this study.

2) Pixel intensity values greater than threshold level were assigned a value of 1 (white; Figure 1, Panel B), and values lower than threshold level were assigned a value of 0 (black).

3) Calculating average cell nucleus area: The command *regionprops *(measures properties for image regions) was then used to sequentially (from left to right of image) label each object at its centroid (Figure 1, Panel C), and to measure area (pixel^2^) of each object in the binary image. The trimmed mean (command *trimmean*) of the constructed area array was calculated with a chosen percent value of 10%. As such, for a given image, 5% of the highest and lowest values from the area cell array were not included in calculation of the mean cell nucleus area. Average cell area (mean ± S.E.M.) at 2.5 ×, 5 ×, and 10 × power was 20.9 ± 0.6 pixels^2^, 62.4 ± 1.1 pixels^2^, and 256.1 ± 18.9 pixels^2^, respectively, and was independent of total number of seeded cells.

4) Determining final cell count: Each value in the area cell array was divided by the average cell nucleus area and rounded to the nearest integer (using command *round*). The sum of the integers then provided the total number of cells for a given image. Final counts were saved as a text file and exported into Microsoft^®^ Excel^®^ software (Version 12.2.8) for column statistics and data organization.

### Algorithm Outputs Compared to Manual Counts

**Figure 2:**
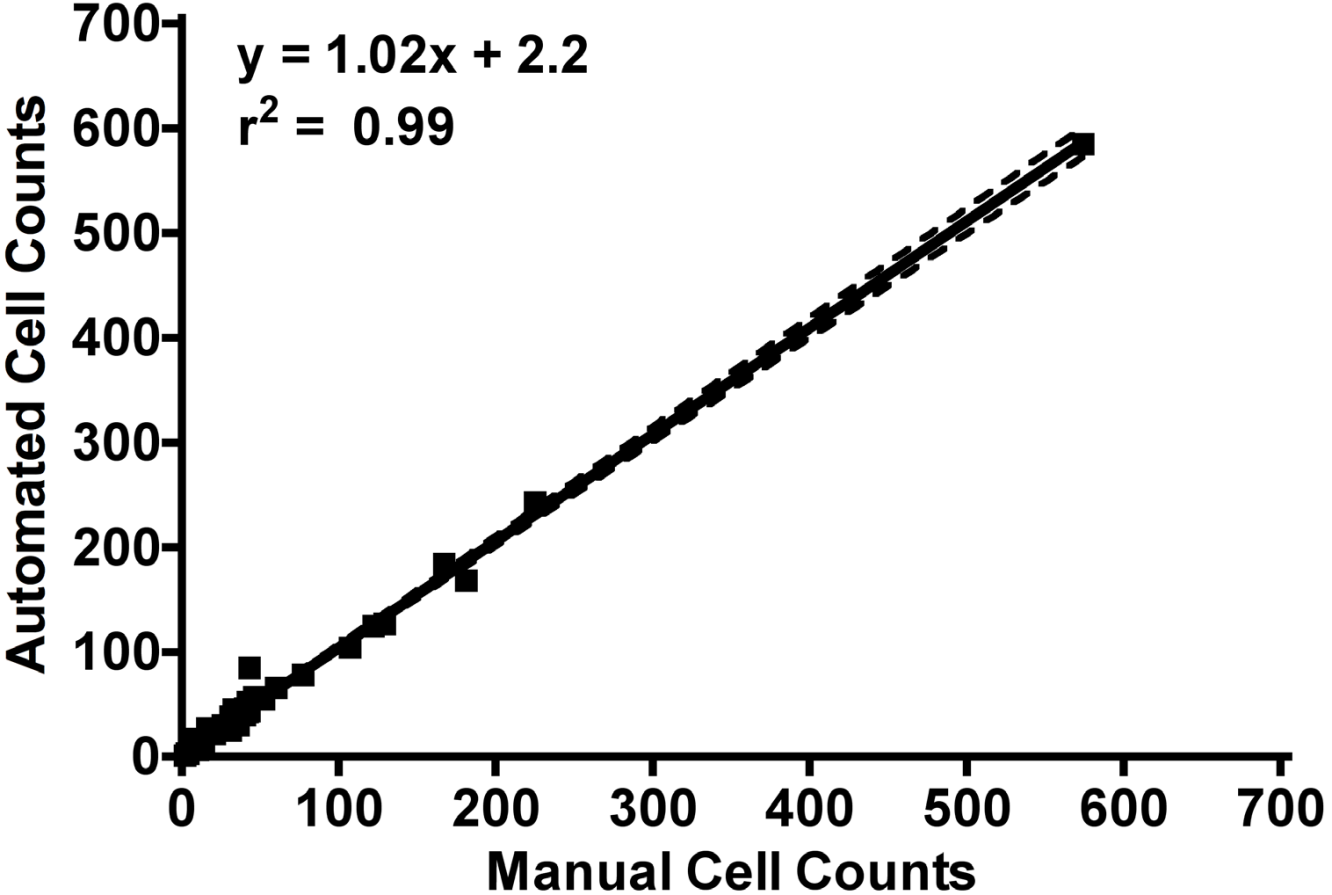
**Manual versus Automated Cell Counts**. Linear regression of manual versus automated cell counts for 47 images, with a correlation of r^2^ = 0.99, P < 0.0001. Dotted lines represent 95% confidence interval for slope and y-intercept.

A blinded experimenter conducted manual cell counts using ImageJ software (ImageJ 1.43u, National Institute of Health, Bethesda, Maryland, USA), which required them to manually place a marker on each cell. A total of 47 images from varying fields of view within each membrane (for 10,000 and 100,000 total seeded cells) were read in as a series of images (.tif series) based on sequential file name order. Using our algorithm, total computing time for 47 images was 14.5 seconds, which was 596 times faster than manual counting [total manual counting time for 47 images = 8640 seconds (2.4 hours)]. Linear correlation between manual and automated cell counts from 47 images images taken using 2.5 ×, 5 ×, and 10 × objectives illustrated near perfect correlation and congruency (r^2^ = 0.99, P < 0.0001, Slope = 1.02, Y intercept = 2.2; Figure 2).

### Algorithm Outputs Compared to Manual Cell Counts Based on Objective Power and Total Seeded Cells

**Figure 3:**
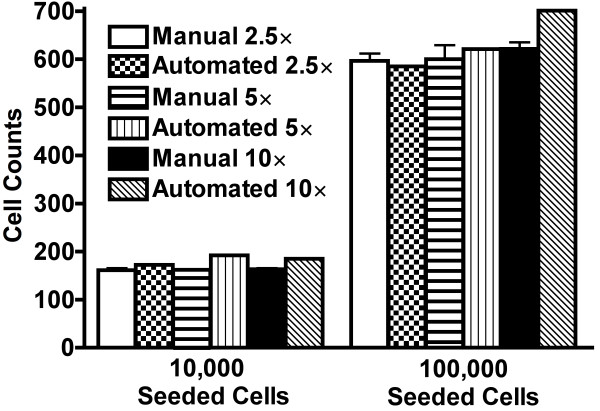
**Manual versus Automated Cell Counts Based on Objective Power**. Comparison of manual versus automated cell counts for varying objective power (2.5 ×, 5 × montages, and 10 × montages) and total seeded cells (10,000 and 100,000 cells). Within each group of total seeded cells, RM ANOVA confirmed no significant difference between manual and automated counts at all objective powers.

To determine the effect of objective power on the software's ability to resolve migrated cells, a single 2.5 × image (centered on the membrane) was captured for experiments that used 10,000 and 100,000 seeded cells, followed by images of the same membrane area at 5 ×, and then 10 ×. Multiple 5 × and 10 × images were assembled into a photomontage (all image resolutions were collected and maintained at 150 dpi) to reconstruct exact membrane area covered by the single 2.5 × image. At each number of total seeded cells (10,000 and 100,000 cells), manual counts (in triplicate) were performed on the single 2.5 × image, 5 × photomontage, and the 10 × photomontage (Figure 3). It is important to note that during cell migration experiments not all seeded cells migrate through the membrane, thus cell counts are lower than the total number of seeded cells.

## Discussion

In the current study, an automated cell counting algorithm was created to quantify resultant migratory 4T1 breast cancer cells in cell migration assays that used 10,000 and 100,000 total seeded cells. The algorithm was only tested in migration assays conducted in modified Boyden chambers using DAPI-stained 4T1 breast cancer cells. For a total of 47 images, automated cell counts had strong correlation with manual counts (r^2^ = 0.99, P < 0.0001; Figure 2). To highlight the congruency between methods, the y-intercept for the linear regression line (Figure 2) indicated that our automated method overestimated an average of only 2 cells under all experimental and imaging conditions. Furthermore, there were no differences in cell counts between methods regardless of experimental conditions (number of cells seeded) or objective power (Figure 3).

Our results support the notion that manual cell counting is time consuming (47 images; manual counting time = 2.4 hours versus automated computing time = 14.5 seconds) and subject to operator bias (hence our experimenter was blinded). Further, it is difficult to reproduce exact measurements using manual methods (hence variability in our manual cell counts, Figure 3), which is likely due to disparate criteria under which manual cell counting is performed from image to image. Our automated method processes and counts all images using set criteria making it immune to the aforementioned sources of error. Upon multiple independent screening of the same 47 images, zero variability was still obtained.

Quantification of migrated cells from cell migration experiments are generally limited to objective powers ranging from 5 × to 200 × [14] and thus require 3 [15] to 10 [16] fields of view in an effort to capture a representative sample of the membrane. The consistency in computed cell counts among different objective magnifications highlights our algorithm's ability to accurately resolve and count cells even under low magnification; thus enabling the user to analyze larger proportions of total membrane surface area in one field of view. This is due to the thresholding portion of our algorithm, which optimizes contrast between cell nuclei and the image background (Figure 1).

Using our algorithm, the number of cells counted depends on the surface area of the majority of nuclei in the image. Therefore, if the majority of cells are multinucleated, then the representative (mean) nucleus area will be greater than if the majority of the cells had single nuclei. We describe (Results - Cell Counting Algorithm section) that our algorithm uses the Matlab^®^ command *round *by which we account for discrepancies in nucleus sizes based on ratios (discrete nucleus surface area/representative nucleus surface area). Under this command, nuclei area ratios of less than 0.5 are discarded and not counted (this accounts for image artifacts, cells that have not completely migrated through pores, cellular debris, etc). Ratios including 1.0 and between 1.0 and 1.5 are counted as single cells, and those including 1.5 and between 1.5 and 2.0 are counted as 2 cells (and so on). Thus, using this ratio and rounding technique our algorithm accounts for biological variations in nuclei size (within a reasonable range). This mathematical approach is definitive, reproducible, and invariable, which is in contrast to the gold standard of manual counting that is dictated by "experience" and "artistic impression".

Irregularities in nuclei shape do not affect how the algorithm calculates average cell nucleus area, as it does not constrain expressed fluorescent area to a specific shape. In other words, assuming surface area is maintained across the majority of cells (as addressed above), fluorescent cell nucleic area is assumed to be independent of shape.

In the current study, using 4T1 cancer cells, we did not observe an abundance of atypical nucleus sizes or shapes. Our research addressed the issue of overlapping cells by using increasing numbers of cells seeded into Boyden chambers (from 10,000 to 100,000 cells). This was based on the reasonable postulate that increased cell number would decrease the distance between migrated cells, thus increasing the opportunity for migrated cells to overlap and be miscounted. We saw no difference in average cell area (mean ± S.E.M.) regardless of total number of seeded cells.

To substantiate the accuracy of our approach, there were no differences in the mean number of cells counted by our algorithm versus those manually counted by a blinded experienced cell biologist (the gold standard). However, repeated manual counts produced intra-observer variability, whereas the counts by our algorithm were devoid of variability.

## Conclusions

We have successfully developed a feasible algorithm for automated cell counting within cell migration assays. Automated cell counts agreed favorably with manual counts for all objective powers and for different levels of total seeded cells. As well, in contrast to manual counting, our automated algorithm counted cells quickly, was independent of bias, and presented zero variability for counting cells multiple times within single images.

Consult http://microvessels.com/cellcounting.html or the corresponding author for access to this software.

## Materials and methods

### Cell Culture

4T1 cells, a gift from Dr. Fred Miller (Wayne State University, Michigan USA), were cultivated in high glucose Dulbecco's Minimal Essential Medium (DMEM) supplemented with 10% sterile FBS. Cells were incubated at 37°C and 5% carbon dioxide. At approximately 80% confluency, cells were washed with HBSS and passaged using 0.25% trypsin-EDTA treatment for dissociation.

### Migration Assays

Migration assays were conducted using a modified Boyden chamber apparatus with a 12-well plate and cell culture inserts with polyethylene terephthalate membranes (8 μm pores, BD Biosciences). 4T1 cells were plated in the upper chamber in serum-free media (10,000 and 100,000 cells). Serum-containing media (10% fetal bovine serum) was added to the bottom chamber as a chemoattractant. After 24 hours of incubation, non-migrated cells were scraped from the top of the membrane with a cotton swab; migrated cells (on the bottom of the membrane) were then fixed in methanol and stained with DAPI. The membranes were mounted on slides, and imaged using fluorescence microscopy at 2.5 ×, 5 × and 10 × magnification (Zeiss Axiovert 200, Zeiss Axiocam HRc camera). Image exposure time was consistent at each magnification (2.5 ×: 581 ms; 5 ×: 206 ms, 10 ×: 49 ms).

### Image Assembly

To reproduce single 2.5 × fields of view at higher objective powers, photomontages (.tif) of overlapping fields of view taken at the 5 × and 10 × power objectives were assembled in Adobe^®^ Photoshop^®^ CS3 (version 10.0.1). No other manipulations or alterations were made to the original images using Photoshop.

### Statistical Analysis and Data Presentation

All data are presented as mean ± S.D., unless stated otherwise. Statistical analysis was performed using Prism Software (version 4, GraphPad Software Inc, La Jolla, CA, USA) and differences were accepted as statistically significant when P < 0.05. Manual versus automated cell counts for all 47 images (Figure 2) were plotted using linear regression analysis. To analyze the effects of objective power and manual versus automated counts within a given level of seeded cells, a repeated measures analysis of variance (RM ANOVA) was conducted (Figure 3).

## Competing interests

The authors declare that they have no competing interests.

## Authors' contributions

All authors have read and approved the final manuscript. BKA generated the Matlab-based algorithm, imaged all cell membranes, contributed to conception and design of the study, analyzed data, drafted the manuscript, and edited the manuscript. PJM carried out migration assay experiments, contributed to conception and design of the study, participated in data analysis and interpretation, and edited the manuscript. NMN performed manual counts on all membranes, participated in data analysis and interpretation, and edited the manuscript. DNJ supervised the project, contributed to conception and design of the study, participated in data analysis and interpretation, and was responsible for final revisions of manuscript.
